# Supplementary material for: Seed bank and growth comparisons of native (Virgilia divaricata) and invasive alien (Acacia mearnsii and A. melanoxylon) plants: implications for conservation
Source: PeerJ. 2018 Aug 21;6:e5466. doi: 10.7717/peerj.5466 (PMC6108313; doi:10.7717/peerj.5466)
Supplement: Supplemental Information 1 [file peerj-06-5466-s001.docx]

## Supplementary S1: Locations (using the Universal Transverse Mercator coordinate system, WGS84 datum) of seed bank sampling sites and representation of habitat types across sampled trees.

| Tree number | Species | Location | Habitat |
| --- | --- | --- | --- |
| 1 | *A. mearnsii* | 33°56.173' S, 022°38.616' E | Forest |
| 2 | *A. mearnsii* | 33°54.693' S, 022°40.055' E | Fynbos |
| 3 | *A. mearnsii* | 33°56.225' S, 022°40.261' E | Disturbed |
| 4 | *A. mearnsii* | 33°58.050' S, 023°03.374' E | Forest |
| 5 | *A. mearnsii* | 34°01.463' S, 023°00.064' E | Disturbed |
| 6 | *A. mearnsii* | 34°01.447' S, 022°59.978' E | Disturbed |
| 7 | *A. mearnsii* | 33°56.461' S, 023°18.788' E | Ecotone |
| 8 | *A. mearnsii* | 33°56.507' S, 023°18.855' E | Ecotone |
| 9 | *A. mearnsii* | 33°56.470' S, 023°17.905' E | Fynbos |
| 10 | *A. mearnsii* | 33°54.713' S, 022°40.401' E | Fynbos |
| 11 | *A. melanoxylon* | 33°56.179' S, 022°38.609' E | Forest |
| 12 | *A. melanoxylon* | 33°54.693' S, 022°40.081 'E | Fynbos |
| 13 | *A. melanoxylon* | 34°01.617' S, 023°10.630' E | Disturbed |
| 14 | *A. melanoxylon* | 34°01.446' S, 023°00.049' E | Fynbos |
| 15 | *A. melanoxylon* | 34°01.438' S, 023°00.054' E | Disturbed |
| 16 | *A. melanoxylon* | 34°01.748' S, 023°10.800' E | Forest |
| 17 | *A. melanoxylon* | 33°55.640' S, 022°40.307' E | Disturbed |
| 18 | *A. melanoxylon* | 33°59.939' S, 022°32.055' E | Ecotone |
| 19 | *A. melanoxylon* | 33°56.633' S, 023°30.725' E | Disturbed |
| 20 | *A. melanoxylon* | 33°56.677' S, 023°30.741' E | Disturbed |
| 21 | *V. divaricata* | 33°54.687' S, 022°40.213' E | Fynbos |
| 22 | *V. divaricata* | 34°02.091' S, 023°11.906' E | Disturbed |
| 23 | *V. divaricata* | 34°01.641' S, 023°10.400' E | Disturbed |
| 24 | *V. divaricata* | 34°01.552' S, 023°00.142' E | Fynbos |
| 25 | *V. divaricata* | 33°54.965' S, 023°08.718' E | Fynbos |
| 26 | *V. divaricata* | 33°54.973' S, 023°08.718' E | Ecotone |
| 27 | *V. divaricata* | 33°54.867' S, 022°40.485' E | Fynbos |
| 28 | *V. divaricata* | 33°53.773' S, 022°39.183' E | Forest |
| 29 | *V. divaricata* | 33°57.492' S, 023°31.158' E | Fynbos |
| 30 | *V. divaricata* | 33°57.354' S, 023°30.857' E | Fynbos |
