# Supplementary material for: Seed bank and growth comparisons of native (Virgilia divaricata) and invasive alien (Acacia mearnsii and A. melanoxylon) plants: implications for conservation
Source: PeerJ. 2018 Aug 21;6:e5466. doi: 10.7717/peerj.5466 (PMC6108313; doi:10.7717/peerj.5466)
Supplement: Supplemental Information 2 [file peerj-06-5466-s002.docx]

## Supplemental S2: General Linear Model (GLM) output tables

Results of GLM exploring the relationship between seedling growth in height and initial height and how this differ between *Virgilia divaricata* (reference species) and *Acacia mearnsii*.*.*

| Effect | Coefficient | p |
| --- | --- | --- |
| Intercept | 4.159 | <0.001 |
| Initial height | -0.512 | <0.001 |
| *Acacia mearnsii* | -3.299 | <0.001 |
| Initial height**Acacia mearnsii* | -0.285 | 0.389 |

Results of GLM exploring the relationship between growth in height and initial height of saplings of *Virgilia divaricata* (reference species), and *Acacia mearnsii* and *A. melanoxylon*.

| Effect | Coefficient | p |
| --- | --- | --- |
| Intercept | 84.045 | <0.001 |
| Initial height | 0.076 | 0.111 |
| *Acacia mearnsii* | -73.842 | <0.001 |
| *Acacia melanoxylon* | -74.331 | <0.001 |
| Initial height**Acacia mearnsii* | 0.226 | <0.001 |
| Initial height**Acacia melanoxylon* | 0.123 | 0.151 |

Results of GLM exploring the relationship between growth in groundline diameter (GLD) and initial GLD of saplings of *Virgilia divaricata* (reference species), and *Acacia mearnsii* and *A. melanoxylon*.

| Effect | Coefficient | p |
| --- | --- | --- |
| Intercept | 0.08 | 0.189 |
| Initial GLD | 0.231 | <0.001 |
| *Acacia mearnsii* | -0.067 | 0.285 |
| *Acacia melanoxylon* | -0.022 | 0.776 |
| Initial GLD**Acacia mearnsii* | -0.004 | 0.868 |
| Initial GLD**Acacia melanoxylon* | -0.105 | 0.034 |

Results of GLM exploring the relationship between growth in biomass index (BI) and initial BI of saplings of *Virgilia divaricata* (reference species), and *Acacia mearnsii* and *A. melanoxylon*.

| Effect | Coefficient | p |
| --- | --- | --- |
| Intercept | 498.989 | 0.026 |
| Initial BI | 0.924 | <0.001 |
| *Acacia mearnsii* | -347.011 | 0.126 |
| *Acacia melanoxylon* | -500.552 | 0.061 |
| Initial BI**Acacia mearnsii* | 0.006 | 0.844 |
| Initial BI**Acacia melanoxylon* | -0.115 | 0.737 |

Results of GLM exploring the relationship between sapling growth (expressed as relative increase in biomass index ‘BI’) of focal saplings and the extent of competition (expressed as summed BI of competitors), and how this differs between *Virgilia divaricata* (reference species) and *Acacia mearnsii* and *A. melanoxylon*.

| Effect | Coefficient | p |
| --- | --- | --- |
| Intercept | 1.682 | <0.001 |
| Competitor biomass | -1.9E^-7^ | 0.943 |
| *Acacia mearnsii* | -0.171 | 0.654 |
| *Acacia melanoxylon* | -0.225 | 0.629 |
| Competitor biomass**Acacia mearnsii* | -3.1E^-6^ | 0.276 |
| Competitor biomass**Acacia melanoxylon* | -2.1E^-6^ | 0.522 |
